# The pivotal role of pyruvate dehydrogenase kinases in metabolic flexibility

**DOI:** 10.1186/1743-7075-11-10

**Published:** 2014-02-12

**Authors:** Shuai Zhang, Matthew W Hulver, Ryan P McMillan, Mark A Cline, Elizabeth R Gilbert

**Affiliations:** 1 Department of Animal and Poultry Sciences, Virginia Tech, Blacksburg, VA USA; 2 Department of Human Nutrition, Foods and Exercise and Metabolic Phenotyping Core, Virginia Tech, Blacksburg, VA USA; 3 3200 Litton-Reaves, Animal & Poultry Sciences Department, Virginia Tech, Blacksburg, VA 24061-0306, USA

**Keywords:** PDC, PDK, Metabolic flexibility

## Abstract

Metabolic flexibility is the capacity of a system to adjust fuel (primarily glucose and fatty acids) oxidation based on nutrient availability. The ability to alter substrate oxidation in response to nutritional state depends on the genetically influenced balance between oxidation and storage capacities. Competition between fatty acids and glucose for oxidation occurs at the level of the pyruvate dehydrogenase complex (PDC). The PDC is normally active in most tissues in the fed state, and suppressing PDC activity by pyruvate dehydrogenase (PDH) kinase (PDK) is crucial to maintain energy homeostasis under some extreme nutritional conditions in mammals. Conversely, inappropriate suppression of PDC activity might promote the development of metabolic diseases. This review summarizes PDKs’ pivotal role in control of metabolic flexibility under various nutrient conditions and in different tissues, with emphasis on the best characterized PDK4. Understanding the regulation of PDC and PDKs and their roles in energy homeostasis could be beneficial to alleviate metabolic inflexibility and to provide possible therapies for metabolic diseases, including type 2 diabetes (T2D).

## Introduction

Maintaining a balance between energy demand and supply is critical for health. Glucose and lipids (fatty acids and ketone bodies), as sources of cellular energy, can compete and interact with each other [1]. The capacity for an organism to adapt fuel oxidation to fuel availability, that is, to preferentially utilize carbohydrate and lipid fuels and to be able to rapidly switch between them is termed metabolic flexibility [2,3]. The failure to match fuel oxidation to changes in nutrient availability is often accompanied by symptoms such as insulin resistance, ectopic lipid accumulation and mitochondrial dysfunction [3,4]. Thus, metabolic inflexibility is tightly related to a series of syndromes such as type 2 diabetes (T2D), obesity, cardiovascular disease and metabolic syndrome.

**Figure 1:**
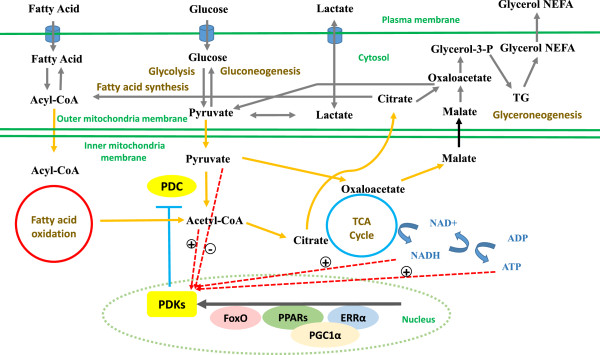
**PDC and PDKs occupy central positions in cellular energy metabolism.** Fatty acid and glucose compete with each other for oxidation at the level of the PDC in mammals. PDC catalyzes the oxidative decarboxylation of pyruvate to form acetyl-CoA and thus links glucose metabolism and fatty acid metabolism. PDC can be phosphorylated by PDKs, which can be regulated by mitochondrial acetyl-CoA, NADH, pyruvate, ATP and nuclear transcription factors. ERRα: Estrogen related receptor α; FoxO: Forkhead box protein O; NEFA: Non-esterified fatty acid; PDC: Pyruvate dehydrogenase complex; PDKs: Pyruvate dehydrogenase kinases; PGC1α: PPARγ co-activator 1α; PPARs: Peroxisome proliferator-activated receptors; TG: Triglyceride; TCA: Tricarboxylic acid.

One of the major enzymes responsible for metabolic flexibility in mammals is the pyruvate dehydrogenase complex (PDC), a mitochondrial multi-enzyme complex that catalyzes the oxidative decarboxylation of pyruvate [5]. PDC controls the conversion of pyruvate, Coenzyme A (CoA) and NAD^+^ into acetyl-CoA, NADH and CO_2_, and thus links fatty acid metabolism, glucose metabolism and the tricarboxylic acid (TCA) cycle [6]. The CoA-activated two-carbon unit produced by the catabolism of pyruvate can be condensed with oxaloacetate in the first reaction of the TCA cycle, or used for fatty acid and cholesterol synthesis [7]. Pyruvate may also be conserved for gluconeogenesis in liver and kidney [8]. Thus, the PDC occupies a central position in cellular energy metabolism (Figure 1).

**Figure 2:**
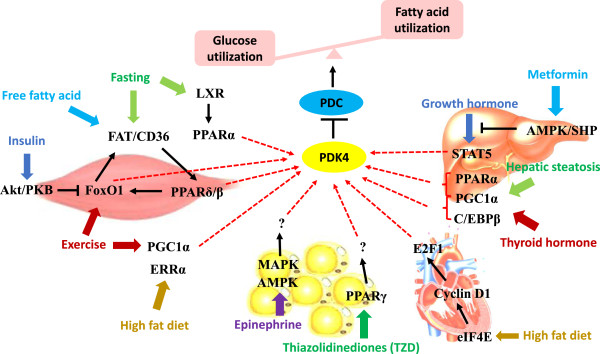
**Transcriptional regulation pathways of PDK4 in different tissues under various nutritional states.** Inactivation of PDC by up-regulation of PDK4 can switch glucose catabolism to fatty acid utilization. There are different transcriptional regulation pathways in skeletal muscle, liver, white adipose tissue and heart under various nutritional conditions (energy deprivation, high fat diet consumption, exercise, diseases, drugs). Akt/PKB: protein kinase B; AMPK: 5’-AMP-activated protein kinase; CD36: Cluster of differentiation 36; C/EBPβ: CCAAT/enhancer-binding protein β; eIF4E: Eukaryotic initiation factor 4E; ERRα: Estrogen related receptor α; FAT: Fatty acid transporter; FoxO1: Forkhead box protein O1; LXR: Liver X receptor; MAPK: p38 mitogen-activated protein kinase; PDC: Pyruvate dehydrogenase complex; PDK4: Pyruvate dehydrogenase kinase 4; PGC1α: PPARγ co-activator 1α; PPARs: Peroxisome proliferator-activated receptors; SHP: Small heterodimer partner; STAT5: Signal transducer and activator of transcription 5.

PDC is more active in the healthy and well-fed state. However, suppression of PDC is crucial for glucose synthesis when glucose is scarce [9]. The inactivation of PDC activity is catalyzed by four highly specific pyruvate dehydrogenase (PDH) kinase (PDK) isozymes that can phosphorylate specific serine residues within the α subunit of the E1 enzyme in the PDC [5,10]. Of all the known isozymes, PDK2 and PDK4 are the most widely distributed and are highly expressed in heart, liver and kidney in humans and rodents. PDK4 is also abundant in pancreatic islets and in skeletal muscles that have high glucose utilization and fatty acid oxidation rates. PDK1 and PDK3 have rather limited tissue distribution [11]. The PDKs activities can be regulated by different levels of metabolites as well as transcription factors under various conditions and in different tissues (Figure 2). Thus the PDC can manage the utilization and storage of fuels to fulfill metabolic flexibility in response to the environment.

This review summarizes the recent studies on PDKs pivotal role in controlling metabolic flexibility, a recent concept in cellular energy metabolism, under various nutritional conditions (energy deprivation, high fat diet consumption, exercise and disease) in diverse tissues (skeletal muscle, liver, white adipose tissue, heart, pancreatic islets and nervous system), with emphasis on the best characterized PDK4. Understanding the regulation of PDKs in different tissues and their roles in energy homeostasis will be beneficial for treatment of different kinds of metabolic diseases.

### PDK and metabolic flexibility in skeletal muscle

As quantitatively the largest organ in the body, skeletal muscle accounts for 30% to 40% of the metabolic rate in adults in the resting state. Contributing to 80% of insulin-stimulated glucose uptake, it is a major site for glucose oxidation and fatty acid metabolism [12]. The skeletal muscle exhibits remarkable metabolic flexibility in fuel usage in response to various metabolic challenges such as energy deprivation and changes in diet composition. In lean healthy individuals, under insulin stimulation, skeletal muscle is able to switch from predominantly lipid oxidation and high rates of fatty acid uptake to the suppression of lipid catabolism and elevated glucose uptake, oxidation and storage [13]. However, obese and T2D patients manifest greater rates of lipid oxidation in skeletal muscle and are relatively insulin resistant, resulting in metabolic inflexibility [13].

#### Energy deprivation

During energy deprivation, glucose is scarce and long-chain fatty acid oxidation is used to meet cellular energy requirements. Reduced food intake and lowered insulin concentrations decrease glucose utilization to conserve glucose [6]. Mammalian PDC activity is suppressed by PDK’s hyper-phosphorylation, limiting the conversion of pyruvate to acetyl-CoA in skeletal muscle [11]. With less acetyl-CoA available, the synthesis of malonyl-CoA, an inhibitor of fatty acid oxidation, is reduced [14]. As a result, fatty acid oxidation is both forced and facilitated by up-regulation of PDK4 [15]. Forty eight hours of fasting in rats (complete food withdrawal) was associated with a 3–4 fold increase in PDK4 protein and mRNA in gastrocnemius muscle, with no effects on PDK2 expression [16]. Forty eight hour-fasted *PDK4* knock-out mice exhibited lowered blood glucose, elevated serum non-esterified fatty acids and greater PDC activity in gastrocnemius muscle [17], consistent with the lower rates of fatty acid oxidation and increased rates of glucose and pyruvate oxidation. However, energy deprivation led to a decrease rather than increase in PDK2 activity in gastrocnemius muscle in *PDK4* knock-out mice [17]. This suggested that PDK2 was not able to compensate for the loss of function of PDK4 in response to fasting. Refeeding the fasted wild-type rats for 48 h reduced *PDK4* mRNA to a level comparable to the control group [16].

The acetyl-CoA and NADH produced by fatty acid oxidation stimulated PDK activity in skeletal muscle [11]. There was also a selective induction of forkhead box O1 (FoxO1) and forkhead box O3 (FoxO3) transcripts in gastrocnemius muscle in mice after 48 h of food withdrawal [18], indicating the involvement of the FoxO in up-regulation of PDK4 in response to short-term changes in nutritional state. PDK4, interacting with fatty acid transporter/cluster of differentiation 36 (FAT/CD36, major fatty acid uptake proteins in muscle), peroxisome-proliferator activated receptor δ/β (PPARδ/β, fatty acid activated nuclear receptor) and FoxO1, provides a framework for regulating muscle fuel preference in response to fasting [19]. During energy deprivation, CD36-facilitated fatty acid flux activates PPARδ/β, which coordinately increases the expression of FoxO1 and PDK4 to inhibit glucose oxidation. The fatty acid flux and decreased insulin concentrations are associated with down-regulation of protein kinase B (Akt/PKB), leading to FoxO1 activation [20]. Since FoxO1 also recruits CD36 to the plasma membrane and induces lipoprotein lipase, all of these enhance fatty acid utilization in skeletal muscle [19]. The novel liver X receptor (LXR)-PPARα metabolostatic signaling axis was also reported to be involved in the muscle energy deprivation response [21,22]. The activation of LXR augmented PPARα signaling to increase fasting-induced up-regulation of PDK4 expression, thus enhancing fatty acid oxidation and decreasing glucose catabolism in skeletal muscle [21,22].

#### Long-term high fat diet consumption

Long-term consumption of a high-saturated fat diet may cause hyperglycemia, hyperinsulinaemia, glucose intolerance and obesity. The administration of a diet high in saturated fats for 4 weeks to rats significantly increased PDK2 and PDK4 protein expression in both fast-twitch white muscle fiber sub-types (anterior tibialis) and slow-twitch red muscle fiber sub-types (soleus) in rats [23]. The slow-twitch red muscle fiber is rich in mitochondria and myoglobin and relies on aerobic metabolism of carbohydrate and lipid fuels. In soleus, the relative increase in PDK4 expression was also linked to a more than 7 fold increase in pyruvate concentration and a 50% reduction in PDC activity compared to that in anterior tibialis [23], indicating greater loss of PDK sensitivity due to pyruvate inhibition in fast-twitch muscle compared to slow-twitch muscle. Consumption of a high fat diet leads to the use of lipid-derived fuels as respiratory substrates in muscle, in part modulated by the up-regulation in PDK activity. The enhanced fatty acid oxidation after feeding high fat diets in slow-twitch muscle is mainly attributed to the up-regulation of PDK4. However, in fast-twitch muscle, increased *PDK2* mRNA was also observed [23], suggestive of a possible coordinate regulation between PDK2 and PDK4 in white muscle fiber sub-types.

PDK4 deficiency leads to inhibition of fatty acid oxidation and increases in glucose oxidation due to greater PDC activity, which increases the conversion of pyruvate into acetyl-CoA. With more acetyl CoA available to synthesize malonyl-CoA, an inhibitor of fatty acid oxidation, the rate of fatty acid oxidation decreases due to a direct feedback loop [14]. However, feeding high-fat diets in the long term neither promotes further ectopic fat accumulation nor worsens insulin resistance [24,25]. After feeding a high-saturated fat diet for 32 weeks in *PDK4* knockout mice, the PDK4 deficient mice also developed hyperinsulinaemia, but less fat accumulation in skeletal muscle and better glucose tolerance as compared to the wild-type mice [25]. The fatty acid synthase activity was also lower, suggesting that the absence of PDK4 may alter signaling components involved in regulation of lipid metabolism [25].

Up-regulation of the orphan nuclear receptor estrogen related receptor α (ERRα) mRNA and protein was found in mice after chronic consumption of a high-fat diet [26]. It has been suggested that PPARγ coactivator 1α (PGC1α) can regulate glucose catabolism and mitochondrial oxidative pathways by increasing PDK4 activity via a PGC1α/ERRα dependent pathway in skeletal muscle [27,28]. ERRα can recruit PGC1α to combine to the *PDK4* promoter and regulate *PDK4* transcription, which is independent of FoxO1 and PPARs [29]. The negative regulation of PDC activity by PDK4 inhibits the entry of pyruvate into the TCA cycle and subsequently blunts cellular glucose oxidation in response to high-fat feeding [26]. Thus PGC1α/ERRα has a key role in high fat diet induced PDK4 up-regulation and metabolic flexibility in skeletal muscle.

#### Exercise

It was found that PDC activation during low to moderate-intensity muscle contraction was ~2 fold higher in *PDK4* knockout mice than in wild-type mice during exercise, regardless of the intensity [30]. *PDK4* mRNA was markedly increased during prolonged exercise and after both short-term high-intensity and prolonged low-intensity exercise in skeletal muscle in mice [31]. The inactivation of PDC in response to both slow-twitch and fast-twitch muscle contraction through up-regulated PDK4 can limit the entry of glycolytic products into the mitochondria for oxidation. The recovery period after exercise also highlights the high metabolic priority of glycogen replenishing to re-establishing the energy homeostasis in skeletal muscle [31]. High fat diet consumption for 18 weeks followed by 12 h exercise also elevated PDK4 expression in skeletal muscle in mice [32], leading to reduced PDC activity and less carbohydrate oxidation. FoxO1 was suggested to be a possible transcription factor related to this change. FoxO1 can sense changes in availability of free fatty acids, and relay this message downstream by modulating transcription of *PDK4*[32]. Un-phosphorylated FoxO1 resides in the nucleus where it can activate transcription of genes that contain insulin response elements. Phosphorylation of FoxO1 through the Akt/PKB pathway leads to nuclear exclusion and destruction [20]. PGC1α also played significant roles in skeletal muscle in response to exercise according to research on horses [33]. PGC1α regulated glucose oxidation while increasing mitochondrial respiration and fatty acid oxidation during post-exercise recovery in Thoroughbred horses [33].

In addition to exercising muscle, during acute endurance exercise in the one-leg cycling model, the resting muscle also showed increased PDK4 expression, likely mediated by elevation in circulating free fatty acids, ligands of PPARs, and up-regulation of *PPAR* pathways [34].

#### Insulin resistance and diabetes

Insulin resistance is mostly characterized as a limited response to stimulated glucose metabolism in skeletal muscle. Also the resistance to suppression of lipid utilization under insulin resistance impaired the capacity to switch between fuels, leading to metabolic inflexibility [13]. This is very common for obese and T2D patients in the insulin simulated condition. Kim et. al induced acute insulin resistance by constant infusion of Intralipid (a fat emulsion) and lactate for 5 h in rats, resulting in 2 to 3 fold higher PDK4 expression in muscle following insulin infusion [35], indicating the impaired ability of insulin to suppress PDK4. The Intralipid and lactate infusion also decreased the phosphorylation of Akt/PKB and FoxO1, illustrating the impaired insulin signaling [35]. A more recent clinical research study showed that growth hormone (GH) can promote lipolysis and reduce insulin sensitivity in human subjects. This was associated with up-regulation of *PDK4* mRNA and decreased active PDC, similar to what is observed during fasting [36]. The research on T2D patients muscle biopsies showed that both *PDK2* and *PDK4* mRNA were increased in comparison to healthy volunteers after overnight fasting [37], which was consistent with the insulin resistance and metabolic inflexibility of T2D patients. Furthermore, methylation status of cytosines in the +160 and +446 region of the *PDK4* promoter was reduced in T2D patients, suggesting that epigenetic modification of mitochondrial genes are involved in regulating substrate switching [37]. However, as one of the transcription factors that regulates expression of PDK4, the *PGC1α* promoter was reported to be hyper-methylated in skeletal muscle of T2D subjects [38] and after overfeeding fat to low birth weight individuals [39], indicating that altered methylation patterns associated with metabolic disease may be promoter specific [37].

Therapeutic interventions have been used to reduce PDK4 expression in diabetes. Beyond insulin, several PDK4 inhibitors were utilized to promote glucose disposal in animal models. Initial studies showed encouraging results with oral administration of dichloroacetate (DCA), but this compound is a weak PDK inhibitor and toxic [40]. More recently, the potent orally administrated drugs such as PDK inhibitors produced by Novartis and AstraZeneca usually include amides of trifluoro-2-hydroxy-2-methylpropionic acid [41]. All of these inhibitors, including the PDK2 inhibitor Nov3r and AZD7545, bind at the lipoyl group binding site of PDK and effectively increase PDC activity [41]. Many drugs target the PDK activity in most peripheral tissues, like DCA [41], but some drugs have better efficacy in specific tissues. For example, AZD7545 elevated PDC activity more effectively in liver than in skeletal muscle and heart and with the loss of efficacy in skeletal muscle of fasted animals [42].

### PDK and metabolic flexibility in liver

One of the primary functions of liver is to regulate the supply of glucose and other metabolic fuels to provide energy to other tissues [20]. The body can balance the blood glucose levels through balancing glucose production and storage in liver and in kidney, and regulating its utilization in peripheral tissues. Under fasting conditions, the liver initially provides glucose from glycogenolysis, the breakdown of liver glycogen stores. With prolonged energy deprivation, the primary glucose source is gluconeogenesis, the synthesis of glucose from non-carbohydrate precursors such as glycerol, lactate and the amino acid alanine [20]. Inactivation of PDC by PDKs can inhibit conversion of pyruvate to acetyl-CoA, resulting in a shift of pyruvate to the TCA cycle or fatty acid synthesis toward gluconeogenesis [43].

Fasting for 48 h did not alter PDC activity in the liver of *PDK4* knockout mice, but intermediates of the gluconeogenic pathway (glucose-6-phosphate, fructose-1,6-bisphosphate, pyruvate, lactate and citrate) were lower [17], indicating a reduced rate of gluconeogenesis and glycolysis. Growth hormone (GH) can increase hepatic PDK4 expression in liver in wild-type mice during fasting through the activation of signal transducer and activator of transcription 5 (STAT5), leading to inhibition of PDC activity, conserving substrates for gluconeogenesis [44]. Metformin, a commonly prescribed drug for T2D, can inhibit GH-induced PDK4 expression via a 5’-AMP-activated protein kinase - small heterodimer partner (AMPK-SHP) dependent pathway to inhibit the combination of STAT5 to the *PDK4* promoter [44].

Hepatic expression of the PDK4 and PDK2, and PDC activity were not affected in wild-type mice fed a high-fat diet for 18 weeks. [24]. High-fat diet feeding induced hepatic steatosis, a condition that occurs when fat accumulation exceeds the oxidation rate [45]. This situation was prevented in *PDK4* knockout mice that consumed a high-saturated fat diet for 32 weeks [25]. This can be explained in part by the altered PGC1α activity in the liver. PGC1α controls expression of gluconeogenic enzymes such as phosphoenolpyruvate carboxykinase (PEPCK). Knocking out *PDK4* could lead to higher levels of PGC1α, consistent with greater activity of PEPCK and a lower capacity for *de novo* fatty acid synthesis [25]. PPARα also showed coordinated regulation with PGC1α in hepatic steatosis, which was demonstrated by the enhanced beneficial effects of clofibric acid, a PPARα agonist, on fatty acid accumulation in *PDK4* knockout mice [46]. In contrast to skeletal muscle, FAT/CD36, key enzymes for fatty acid transport, were not involved in the reduced fat accumulation in liver [25].

Under diabetic conditions, expression of the PDK genes, particularly PDK4, are significantly elevated in the liver, which could help explain the increased rates of gluconeogenesis [47], and the beneficial effects of metformin. Research on the diabetic mice model that is deficient in hepatic insulin receptor substrates 1 and 2 (IRS 1/2) revealed that both knockdown and knockout of the *PDK4* gene led to improvement of glycemic control and glucose tolerance. PDK4 was more efficient in regulating metabolic flexibility than PDK2 in liver [47]. Combined with the results from the other studies, it seems that PDK2 mainly regulates glucose utilization whereas PDK4 may be involved in both system glucose metabolism and hepatic gluconeogenesis.

Thyroid hormone (T_3_) controls multiple aspects of hepatic energy metabolic processes, such as fatty acid oxidation, lipogenesis and glucose oxidation. Experimental hyperthyroidism can induce PDK4 expression in the liver [48,49], skeletal muscle [50] and heart [51], leading to inhibition of PDC activity. Two binding sites for thyroid hormone receptor β were identified in the promoter of the rat *PDK4* gene [52]. Besides functioning as a T_3_ co-activator, PGC1α can also enhance the T_3_ induction of hepatic PDK4 expression in rats [52]. The CCAAT/enhancer-binding protein β (C/EBPβ), as a transcription factor for genes encoding gluconeogenic enzymes such as PEPCK, also stimulates hepatic *PDK4* expression in rats through two C/EBPβ-response elements in the *PDK4* promoter and also participates in the T_3_ induction of *PDK4* transcription [53].

### PDK4 and metabolic flexibility in white adipose tissue

Compared to the skeletal muscle and liver, relatively little research is reported on metabolic flexibility in white adipose tissues (WAT). WAT is a crucial organ for a fatty acid metabolic process referred as adipocyte glyceroneogenesis. This pathway uses pyruvate, alanine, glutamine or any substances from the TCA cycle as precursors to synthesize dihydroxyacetone phosphate (DHAP) and finally to produce glycerol-3-phosphate (G3P) for triacylglycerol (TAG) synthesis [54]. PDC is linked to this process and suppression of PDC allows increased use of lactate and pyruvate for glyceroneogenesis [55].

As an activator of glyceroneogenesis, thiazolidinediones (TZD) increased *PDK4* mRNA expression in subcutaneous, periepididymal and retroperitoneal WAT depots in *fa/fa* Zucker rats, a genetic obese, insulin resistant model, while *PDK2* mRNA was not affected, indicating the vital role of PDK4 in glyceroneogenesis. TZD-induced PDK4 expression was tissue-specific because liver and muscle did not respond to such a treatment [56]. Similar results were observed for 3 T3-F442A adipocytes *in vitro*, using PDK4 inhibitors, DCA and leelamine, and *PDK4* siRNA. Both 500 μmol/L DCA and 50 μmol/L leelamine inhibited pyruvate incorporation into triglycerides. Incorporation of [1-^14^C] pyruvate into lipids was reduced 40% after transfection of adipocytes with *PDK4* siRNA [56]. PPARγ is a nuclear receptor regulated by the insulin-sensitizing TZDs. PDK4 is an indirect target of PPARγ. Thus the regulation of PDK4 by TZD in WAT tightly relates to PPARγ [57].

Apart from TZD, acute epinephrine treatment also increased *PDK4* mRNA through p38 mitogen-activated protein kinase (*MAPK*) and *AMPK* pathways in cultured adipocytes [58] and in epididymal WAT depots in obese, insulin resistant rat models that were induced by high fat diets [59]. *PDK2* mRNA was still unaffected. Two hours of swimming produced similar results as epinephrine treatment in WAT in both lean and obese rats [59]. Combined with increased G3P synthesis via PEPCK, more glyceroneogenesis allows increased re-esterification of non-esterified fatty acids into TAG from lipolysis, while glucose oxidation is reduced in these adipocytes [58]. With a major role in glucose clearance and fat synthesis/storage, up-regulation of PDK4 during exercise, epinephrine and TZD treatment leading to PDC inhibition, promotes energy storage in WAT. More work is needed for elucidating the transcriptional pathways involved in PDK4 up-regulation in WAT [60].

### PDK4 and metabolic flexibility in heart

Metabolic inflexibility always accompanies cardiomyopathy, particularly during ischemia, and may even cause heart failure [41]. Failure to oxidize enough carbohydrate to meet energy demands is an important reason for cardiac inefficiency. This can be demonstrated by cardiac-specific overexpression of PDK4, which is sufficient to cause a loss of metabolic flexibility and exacerbate cardiomyopathy [61]. Overexpression of PDK4 in heart with a transgenic mice model was associated with a decrease in glucose catabolism and a corresponding increase in fatty acid oxidation. This transgenic model also expressed a constitutively active form of the phosphatase calcineurin, and thus caused hypertrophy in cardiomyocyte fibrosis and a striking increase in mortality [61].

In mice that were fed a high fat diet for 10 days, the cardiac carbohydrate oxidation markedly decreased, with up-regulation of PDK4 activity. The high fat diet induced cardiac metabolic alterations through the eukaryotic initiation factor 4E (*eIF4E*)/*cyclin D1*/*E2F1/PDK4* pathway [62].

During moderately severe ischemia, free fatty acids are the primary fuel in mitochondrial oxidation [43]. While glycolysis is still active and glucose is used for lactate production to yield ATP, independent of oxygen, the inactivation of PDC facilitates fatty acid use. Ischemia causes pyruvate to be converted to lactate, thereby increasing the acidification within the myocardium [41]. Thus inhibition of PDK activity by DCA is vital to increase the ATP production as well as the Ca^2+^ uptake, and use of the combination of glucose-insulin-K^+^ or fatty acid oxidation inhibitors are also beneficial [41].

Angiotensin II (Ang II), the main effector in the renin angiotensin system in heart failure, can induce marked cardiac insulin resistance, leading to the cardiac metabolic switch from glucose to fatty acid oxidation, producing metabolic inflexibility and cardiac inefficiency [63]. PDK4 is highly expressed in this Ang II induced hypertrophy model and deletion of PDK4 prevents the Ang II induced reduction in glucose oxidation and prevents diastolic dysfunction [63]. Inhibition of PDK4 activity has become a new therapeutic strategy against heart disease [41].

### PDK and metabolic flexibility in the central nervous system

The brain also takes advantage of glucose oxidation as the primary energy source. Cultured astrocytes expressed more PDK2 and PDK4 compared to neurons, consistent with the lower PDH activity and higher lactate production displayed by cultured astrocytes [64]. There is accumulating evidence that alterations in PDKs activity are linked to the development of several neurological disorders. For instance, Alzheimer’s disease was associated with dysfunction in PDH activity and glucose metabolism [65]. Brain aging is associated with reduced *PDK1* and *PDK2* mRNA in the cerebellum and elevated *PDK2* mRNA in the hippocampus and cerebral cortex [66], and *PDK2* mRNA up-regulation was involved in glioblastoma [67].

Hypothalamic neurons are sensitive to nutritional signals and can regulate energy balance and glucose homeostasis. However, the underlying complex mechanisms are still not completely understood. Recent studies on mice fasted for 48 h revealed a gene expression profile in hypothalamus consistent with reduced glucose utilization and increased lipid oxidation, including elevated *PDK4* mRNA, consistent with the results in skeletal muscle, liver, heart and kidney [68]. The up-regulation of PDK4 was also observed in hypothalamus during neonatal rat fasting for 6 h, reflecting an attempt to conserve energy during neonatal food deprivation [69]. This also indicates that the neonatal brain is not spared from glucose restriction during energy crisis, but instead the neonatal brain can use ketones derived from fatty acid metabolism as the major source of energy [69]. However, only limited studies are reported for PDK’s effect on hypothalamic energy balance. More research is expected.

### PDK and metabolic flexibility in other tissues

#### Pancreatic islets

In murine pancreatic β cells, both high fatty acid and high glucose treatment increased PDK activity and decreased PDH activity. Palmitate up-regulated mRNA expression of *PDK1*, *PDK2* and *PDK4*, while high glucose increased *PDK1*, *PDK2* mRNA but reduced *PDK4* mRNA [70], suggestive of different transcriptional regulation. Thus the induction of PDK expression by both glucose and fat accompanies the decline in β cell metabolism flexibility during the progression from obesity to T2D [70].

Chronic exposure to hyperglycemic conditions results in glucotoxicity in β cells. Glucotoxicity impairs glucose simulated insulin secretion (GSIS), contributing to the development of T2D. Metabolomic analysis of β cells after exposure to high glucose (25 mM for 20 h) revealed an increase in glucose and decrease in fatty acids during GSIS, but no significant changes in PDK2 protein [71]. Similar research on insulinoma E (INS-1E) β cell lines showed an increase in PDC E1α subunit phosphorylation during high glucose treatment (50 mM for 48 h). Knockdown of *PDK1* and *PDK3* led to a marked reduction in PDC inactivation. However, the PDC inactivation was not associated with altered GSIS [72]. It is possible that PDC activity in INS-1E β cells is in excess and therefore lowering its activity is of little consequence. Prolactin can also induce GSIS in INS-1E cell lines by suppression of PDKs and increased PDC activity, suggestive of a novel role for lactogens in diabetes treatment [73]. As the most important organ involved in the pathogenesis of T2D, more research on metabolic flexibility in pancreatic β cells is needed.

#### Cancer cells

Cancer cells have a unique way to acquire energy, termed the Warburg effect. They utilize increased glycolysis and suppress mitochondrial glucose oxidation to provide energy with a proliferative advantage, conducive to apoptosis resistance and even increased angiogenesis [74]. In low nutrient conditions, the Warburg effect was enhanced through a mechanism involving reactive oxygen species (ROS)/AMPK- dependent activation of PDK [75,76]. PDK1 and PDK3 are the main isoforms related to the Warburg effect [41]. Thus inhibition of PDK with either small interfering RNAs or orphan drugs, such as DCA, can shift the metabolism of cancer cells from glycolysis to glucose oxidation, and may provide a powerful approach to treat cancer [77].

## Conclusions

As the major fuels for substrate oxidation to provide energy in mammals and human beings, glucose and fatty acids can compete with each other at the level of the PDC. PDC is normally active in most tissues in the well-fed state. However, suppression of PDC activity by PDKs is crucial to provide pyruvate and other three-carbon compounds for glucose synthesis when glucose is in demand. PDKs play a pivotal role in stable substrate switching and energy homeostasis, which is known as metabolic flexibility, especially under some extreme nutrient conditions.

The alteration of PDKs expression, particularly PDK4, has a tremendous influence on the health status of the organism. Inhibition of PDK4 can benefit specific tissues, such as skeletal muscle, liver and heart, but up-regulation of PDK4 is beneficial in white adipose tissue in obese and insulin resistant rodents. This can be due to tissue-specific physiology. Glucose is the major fuel source for most tissues, inhibition of PDK4 thereby leading to PDC activation and more glucose oxidation. As the major lipid storage organ, white adipose tissue can take advantage of fatty acids as an energy source, thus increased glyceroneogenesis caused by up-regulated PDK4 is beneficial. The tissue-specific physiology also involves diverse transcriptional regulation pathways, involving transcription factors such as FoxO and PPARs (Figure 2).

Metabolic inflexibility, combined with abnormal PDKs activity, is directly associated with many diseases, such as T2D, obesity, metabolic disorders, cardiomyopathy, neurological disorders and several cancers. Future research on PDC and PDKs regulation in various conditions and different tissues will be beneficial to alleviate metabolic inflexibility and to provide possible therapies for numerous diseases.

## Abbreviations

Akt/PKB: Protein kinase B; AMPK: 5’-AMP-activated protein kinase; Ang II: Angiotensin II; CD36: Cluster of differentiation 36; C/EBPβ: CCAAT/enhancer-binding protein β; DCA: Dichloroacetate; DHAP: Dihydroxyacetone phosphate; eIF4E: Eukaryotic initiation factor 4E; ERRα: Estrogen related receptor α; FAT: Fatty acid transporter; FoxO: Forkhead box protein O; G3P: Glycerol-3-phosphate; GH: Growth hormone; GSIS: Glucose simulated insulin secretion; IRS 1/2: Insulin receptor substrates 1 and 2; LXR: Liver X receptor; MAPK: p38 mitogen-activated protein kinase; PDC: Pyruvate dehydrogenase complex; PDH: Pyruvate dehydrogenase; PDK: Pyruvate dehydrogenase kinase; PEPCK: Phosphoenolpyruvate carboxykinase; PGC1α: PPARγ co-activator 1α; ROS: Reactive oxygen species; PPARs: Peroxisome proliferator-activated receptors; SHP: Small heterodimer partner; STAT5: Signal transducer and activator of transcription 5; T2D: Type 2 diabetes; T3: Thyroid hormone; TAG: Triacylglycerol; TCA: Tricarboxylic acid; TZD: Thiazolidinediones; WAT: White adipose tissue.

## Competing interests

The authors declare that they have no competing interests.

## Authors’ contributions

SZ searched the literature, selected relevant studies and drafted the manuscript. ERG, MWH and MAC edited the manuscript. All authors have read and have given final approval of the version to be published.
